# Pancreatic Acinar Cell Carcinoma: A Rare Pancreatic Malignancy with Distinct Biology and Emerging Therapeutic Opportunities

**DOI:** 10.3390/cancers18142315

**Published:** 2026-07-17

**Authors:** Noor Farhoud, Raed Moh’d Taiseer Al-Rajabi, Joaquina Celebre Baranda, Haoran Li, Wei Zhang, Ravi Kumar Paluri, Ashish Manne, Prasad Dandawate, Weijing Sun, Anup Kasi

**Affiliations:** 1 Department of Internal Medicine, University of Kansas Medical Center, Kansas City, KS 66160, USA; 2 Department of Internal Medicine, Division of Medical Oncology, University of Kansas Medical Center, Westwood, KS 66205, USA; 3 Department of Hematology and Oncology, Wake Forest School of Medicine, Winston-Salem, NC 27157, USA; 4 Department of Internal Medicine, The Ohio State University Comprehensive Cancer Center, Columbus, OH 43210, USA; 5 Department of Cancer Biology, University of Kansas Medical Center, Kansas City, KS 66160, USA

**Keywords:** pancreatic acinar cell carcinoma, pancreatic cancer, acinar cell carcinoma, molecular profiling, gene fusions, *BRAF* fusion, homologous recombination deficiency, targeted therapy, immunotherapy, precision oncology, pancreatic neoplasms

## Abstract

Pancreatic acinar cell carcinoma (PACC) is a rare exocrine pancreatic malignancy accounting for approximately 1–2% of adult pancreatic neoplasms. Although historically grouped with pancreatic ductal adenocarcinoma (PDAC), accumulating evidence demonstrates that PACC represents a biologically distinct entity with unique clinical, pathologic, and molecular characteristics. Compared with PDAC, PACC more commonly presents as a large pancreatic mass, is less frequently associated with obstructive jaundice, and exhibits lower rates of *KRAS* mutations. Recent genomic studies have identified recurrent alterations involving DNA damage repair pathways, WNT/β-catenin signaling, and actionable kinase fusions, including *BRAF* and *RAF1* rearrangements. In advanced disease, fluoropyrimidine- and platinum-based regimens appear to demonstrate greater activity than traditional gemcitabine-based approaches, although prospective comparative data are lacking. This review summarizes the current understanding of the epidemiology, clinical presentation, pathology, molecular landscape, treatment approaches, and prognostic factors associated with PACC. We highlight emerging opportunities for precision oncology and discuss ongoing challenges in the management of this uncommon pancreatic malignancy.

## Simple Summary

Pancreatic acinar cell carcinoma is a rare exocrine pancreatic malignancy with clinical, pathologic, and molecular features that distinguish it from pancreatic ductal adenocarcinoma, the most common form of pancreatic cancer. Due to its rarity, many aspects of its diagnosis and treatment remain poorly understood, and most available evidence comes from small studies. This review summarizes current knowledge about how pancreatic acinar cell carcinoma presents, its unique biological and genetic features, available treatment approaches, and factors that influence patient outcomes. Recent research has shown that this cancer often contains specific genetic alterations that may be targeted with personalized therapies, creating new opportunities for treatment. By bringing together the latest clinical and molecular evidence, this review aims to improve awareness of this rare disease, support more informed treatment decisions, and highlight areas where future research may lead to better outcomes for patients.

## 1. Introduction

Pancreatic acinar cell carcinoma (PACC) is a rare exocrine malignancy with a reported incidence ranging from 0.2% to 4.3% of adult pancreatic neoplasms, most commonly cited as approximately 1–2% in large surgical series [1,2,3,4]. Population-based analyses demonstrate that the incidence of PACC increased by approximately 73% between 2005 and 2015, significantly outpacing the 22% rise observed for PDAC over the same period (*p* < 0.01) [5].

Clinically, PACC most commonly presents in middle-aged to older adults and is frequently characterized by large tumor size at diagnosis [1,6,7,8,9]. Although surgical resection remains the cornerstone of management for localized disease, recurrence rates remain substantial, particularly among patients with adverse pathologic features such as nodal involvement [10,11].

Molecular profiling has further distinguished PACC from PDAC, revealing a low rate of *KRAS* mutations and recurrent alterations in DNA repair pathways, WNT/β-catenin signaling, and chromosomal instability genes [12,13,14,15,16,17,18,19].

Given its rarity, most available data derive from retrospective series and small cohort studies. Consensus regarding optimal systemic therapy remains limited. Nevertheless, emerging genomic studies and increasing use of molecularly guided treatment strategies are improving management.

In this review, we synthesize current evidence regarding the clinical presentation, molecular characteristics, treatment approaches, recurrence patterns, and prognostic factors of PACC. We aim to clarify how this entity differs from PDAC and to highlight evolving opportunities for more informed management.

This narrative review was conducted through a systematic search of PubMed/MEDLINE and Embase without date restrictions, using the terms ‘pancreatic acinar cell carcinoma,’ ‘acinar cell carcinoma of the pancreas,’ ‘PACC,’ and ‘acinar carcinoma treatment.’ Reference lists of identified articles were reviewed for additional sources. Pan-cancer genomic analyses and basket trial data were included where PACC-specific data were limited. No language restrictions were applied.

## 2. Clinical Presentation

PACC is a rare malignancy most commonly presenting in older adults, with a median age at diagnosis of approximately 60–65 years across contemporary series [1,2,3,4,20]. Recent population-based data report a median age of 64.7 years with a male predominance of 70.5% [21]. Studies consistently demonstrate a younger presentation compared with PDAC (e.g., 50.8 ± 10.9 vs. 59.4 ± 10.9 years, *p* < 0.001) [20]. A male predominance is reported in most cases [7,22,23].

### 2.1. Symptoms and Clinical Features

Clinical manifestations are often nonspecific and generally less severe than those observed in PDAC. Abdominal pain is the most common presenting symptom, reported in approximately 40–60% of patients, followed by weight loss (15–45%) and back pain (up to 50%) [3,4,7]. Jaundice is relatively uncommon (7–20%) compared to PDAC, even in tumors located in the pancreatic head, likely reflecting the expansile growth pattern of PACC, which tends to displace rather than infiltrate the bile duct [7,8,9].

A substantial subset of patients presents with advanced disease at diagnosis. Across studies, 33–50% of patients have distant metastases at presentation, with the liver representing the most common site (approximately 35–80% of metastatic cases) [10,22,24]. Less common metastatic sites include lung, peritoneum, bone, skin, and brain [22,25]. Notably, hepatic metastases in PACC tend to be larger than those observed in PDAC (median 6.7 cm vs. 1.2 cm in one comparative study, *p* < 0.001) [20].

Rarely, PACC may manifest with lipase hypersecretion syndrome, characterized by subcutaneous fat necrosis (panniculitis), polyarthralgia, and elevated serum lipase levels. This syndrome occurs in a small minority of cases, whereas asymptomatic lipase elevations appear to be more common [7,22,26].

### 2.2. Laboratory Findings

Serum tumor markers are often unhelpful in PACC. CA19-9 is elevated in only 10–30% of patients, with median values substantially lower than in PDAC (e.g., 16.9 U/mL vs. 280.1 U/mL, *p* < 0.001) [20,26]. Similarly, carcinoembryonic antigen (CEA) levels are typically normal or only mildly elevated [9,26]. Alpha-fetoprotein (AFP) elevation has been reported in a minority of patients [9]. Lower total bilirubin levels and lower neutrophil-to-lymphocyte ratios have also been described compared with PDAC [1].

### 2.3. Tumor Location and Size

PACC may arise throughout the pancreas, with distribution roughly even between the head and body/tail [22,24,27,28,29,30]. Compared with PDAC, PACC demonstrates a higher relative frequency in the body and tail (up to 61.5% vs. 36.6% in PDAC, *p* = 0.009) [1].

Tumors are typically large at diagnosis. Median tumor size ranges from 4.0 to 5.8 cm, with reported means up to 7–8 cm in surgical cohorts [3,4,11,17]. Tumors exceeding 10 cm are not uncommon, reported in approximately 10–20% of patients [9,20].

### 2.4. Radiographic Features

On cross-sectional imaging, PACC most commonly appears as a large, well-circumscribed mass with heterogeneous enhancement, reported in up to 79.6% of cases in the largest imaging series [27,31]. Exophytic growth and internal necrosis or cystic degeneration are characteristic features described across multiple series, with cystic or necrotic components reported in 60–80% of cases [27,28,29,32,33]. A partially enhancing capsule-like rim has been described, particularly on MRI [29]. Biliary ductal dilatation is uncommon, present in fewer than 15% of cases even with head-of-pancreas lesions [27]. Imaging features associated with worse survival include T1 hyperintensity, hypoattenuating necrotic components, and splenic vein invasion [27].

## 3. Histopathology

### 3.1. Gross Features

PACC is typically a large, well-circumscribed, and fleshy neoplasm, often measuring between 5 and 11 cm at presentation [6]. Tumor size at presentation is substantially larger compared with PDAC [3,4,11]. On gross examination, tumors are soft, tan to red, and frequently demonstrate areas of hemorrhage and necrosis. Unlike PDAC, PACC characteristically lacks a prominent desmoplastic stromal reaction, a feature that contributes to its soft consistency [6,34].

### 3.2. Microscopic Morphology

PACC is highly cellular and demonstrates a spectrum of architectural patterns, often within the same tumor. The most common growth patterns are acinar and solid, each constituting the predominant component in approximately 30–50% of cases, while the remaining tumors exhibit a mixture of these patterns [6,34,35]. Less frequently observed patterns include glandular, trabecular, gyriform, cystic, or intraductal growth [6,10]. Rare morphologic variants such as spindle cell change or oncocytic differentiation have also been described [10].

Characteristic acinar formations containing pinpoint lumina resemble normal pancreatic acini and, when present, serve as an important diagnostic clue [10]. Tumor cells are typically arranged in nests, cords, or solid sheets [10]. The neoplastic cells have moderate amounts of eosinophilic, granular cytoplasm reflecting cytoplasmic zymogen granules [6,10]. Nuclei are round to oval, often basally oriented, with mild to moderate atypia and a single prominent nucleolus [24,34,36]. These features can be particularly helpful in less well-differentiated tumors [34].

Mitotic activity is variable, and the proliferative index (Ki-67) is frequently elevated, with reported mean values around 20–30% but ranging widely from low to high-grade proliferative activity [9,36]. Necrosis is common, reported in more than half of cases [6,10,34]. Vascular invasion is frequent, whereas perineural invasion is less common [10].

### 3.3. Special Stains

Periodic acid–Schiff (PAS) staining following diastase digestion reveals diastase-resistant cytoplasmic granules in over 90% of tumors, supporting acinar differentiation [8,35]. Histochemical staining for enzymatically active lipase (butyrate esterase) is positive in approximately 70–75% of cases but is not routinely performed in practice [8].

### 3.4. Immunohistochemical Profile

Immunohistochemistry plays a central role in confirming acinar differentiation. The most sensitive and specific markers are pancreatic exocrine enzymes. Trypsin is positive in approximately 95–100% of cases and remains the most widely used diagnostic marker. Chymotrypsin is also frequently positive, although with variable sensitivity [6,10,22,35]. Lipase is less consistently expressed, and amylase has low sensitivity and limited diagnostic utility [10,22]. More recently, antibodies recognizing the COOH-terminal portion of BCL10 (which cross-reacts with carboxyl ester lipase, CEL) have emerged as highly sensitive markers, demonstrating positivity in the majority of tumors, including occasional trypsin-negative cases [22]. These findings support the combined use of trypsin and BCL10 as the most reliable diagnostic markers in small biopsy specimens [22].

### 3.5. Cytokeratin Expression

PACC typically expresses low molecular weight cytokeratins, including CK8 and CK18, and is positive for broad-spectrum cytokeratin markers such as AE1/AE3 and CAM5.2 [34,36]. Although PACC was historically considered negative for ductal cytokeratins, more recent studies have demonstrated CK19 expression in up to 80% of cases and CK7 expression in approximately 50–70% [10]. Importantly, CK7 or CK19 positivity does not exclude the diagnosis of PACC although they are typically markers of ductal carcinoma [10,22]. Some studies have suggested that CK19 expression may correlate with more aggressive clinical behavior, although this requires validation in larger cohorts [22].

### 3.6. Neuroendocrine Differentiation

Focal neuroendocrine differentiation is common, with scattered tumor cells (typically <25–30%) demonstrating immunoreactivity for chromogranin or synaptophysin in approximately one-third to one-half of cases [6,24,34,36]. When more than 30% of tumor cells express neuroendocrine markers, mixed acinar–neuroendocrine carcinoma classification should be considered [34]. True mixed acinar–ductal or mixed acinar–neuroendocrine–ductal carcinomas have also been described, provided each component comprises a substantial proportion of the tumor [24,34].

### 3.7. Cytologic Features and Diagnostic Pitfalls

PACC is frequently misdiagnosed on cytology, most commonly as pancreatic neuroendocrine tumor or PDAC. Distinguishing features include the absence of significant nuclear pleomorphism characteristic of poorly differentiated PDAC, lack of desmoplastic stroma, and strong expression of exocrine enzyme markers [37]. These diagnostic challenges highlight the importance of performing acinar enzyme immunostains in suspicious cases.

## 4. Molecular Findings

### 4.1. Distinction from PDAC

PACC is genomically distinct from PDAC, as several canonical PDAC driver mutations occur at much lower frequencies [38]. Potentially actionable molecular alterations and their corresponding therapeutic strategies are summarized in Table 1. Activating *KRAS* mutations are detected in approximately 1–4% of pure PACC cases, in contrast to >90% of PDACs [12,13,14]. *TP53* mutations are observed in roughly 10–15% of primary PACC tumors, with higher frequencies reported in metastatic lesions [12,35]. Genomic alterations in *CDKN2A* and *SMAD4*/DPC4 occur in a minority of cases and are far less frequent than in PDAC [15,35]. Likewise, loss of DPC4 protein expression, a common event in PDAC, is very rare in PACC [16,39].

### 4.2. Chromosomal Instability

PACC exhibits significant chromosomal instability with widespread copy number alterations [16]. Frequent chromosomal losses include 1p, 3p, 4q, 5q, 6q, 8p, 9p, 11p, 13q, 16q, 17p, and 18q, while recurrent gains involve 1q, 7, 8q, 12, 17q, 19p, and 20q [16,46,47]. One of the most common alterations is loss of chromosome 11p, occurring in about 50% of tumors [16].

Loss of 18q correlates with reduced or absent expression of deleted in colorectal carcinoma (DCC) protein and may contribute to tumor progression [46]. Amplification of c-MYC at 8q24 has been reported in approximately 15–20% of PACC cases, although its role in biological aggressiveness remains incompletely defined [46].

### 4.3. Wnt/β-Catenin Pathway Alterations

Alterations in the Wnt/β-catenin signaling pathway are also relatively common in PACC. Mutations in *APC* or *CTNNB1* occur in approximately 10–25% of tumors, and abnormal nuclear accumulation of β-catenin is observed in a subset [15,16,17,18]. Importantly, *APC* inactivation often occurs through chromosomal loss and promoter hypermethylation rather than point mutations, with *APC* loss and methylation reported in nearly half of tumors [18]. Reduced *APC* mRNA and protein expression correlate with copy number alterations, and similar changes have been observed in adjacent non-neoplastic pancreatic tissue, suggesting a possible “field cancerization” phenomenon [18,48]. Field cancerization refers to the concept that molecularly altered but histologically normal-appearing tissue adjacent to a tumor harbors early genomic changes that predispose it to neoplastic transformation. In the context of PACC, this observation raises questions about whether acinar cells in the surrounding pancreas represent a precancerous field, with potential implications for surveillance strategies and the biological origin of multifocal disease, though prospective clinical data on this question remain limited [17,18]. Alterations in the *APC*-β catenin pathway may help explain the observed activity of chemotherapeutics such as 5-fluorouracil, irinotecan, and oxaliplatin in some retrospective series, although this therapeutic link remains inferential rather than established [19].

### 4.4. DNA Damage Repair and Homologous Recombination Deficiency

One of the most clinically relevant molecular features of PACC is the high prevalence of alterations in DNA damage repair (DDR) and homologous recombination repair (HRR) genes. Germline or somatic mutations in *BRCA2*, *BRCA1*, *PALB2*, *ATM*, *CHEK2*, and other Fanconi anemia pathway genes are reported in approximately 15–40% of cases, particularly in tumors with pure acinar histology [35,38,40,44,49]. Large pan-cancer analyses have demonstrated that PACC harbors one of the highest rates of germline *BRCA2* pathogenic variants among solid tumors [40,41]. Contemporary molecular profiling studies continue to demonstrate recurrent alterations involving *BRCA2*, *CDKN2A*, and *SMAD4* [50]. These findings have potential therapeutic relevance, as tumors with biallelic inactivation of Fanconi anemia pathway genes may be sensitive to platinum agents and PARP inhibitors by analogy with BRCA-associated malignancies [42,50]. However, direct evidence from prospective PACC trials is lacking, and these recommendations are based on extrapolation from other tumor types.

Mutational signatures associated with DNA repair defects have been reported in about 70% of cases. For example, mutational signature 3 has been detected and has been attributed to *BRCA1* and/or *BRCA2* loss [51].

### 4.5. Gene Rearrangements and MAPK Pathway Activation

Gene fusions are particularly enriched in *KRAS*-wild-type pancreatic neoplasms, a molecular characteristic that distinguishes PACC from PDAC and may contribute to the high prevalence of actionable kinase fusions observed in this disease [52]. Recurrent gene rearrangements that activate the MAPK signaling pathway represent a major molecular feature of PACC. Recent large-scale genomic analyses suggest that oncogenic fusions may represent a dominant class of actionable alterations in PACC, further emphasizing the importance of RNA-based sequencing and fusion detection [53]. Among these alterations, *BRAF* and *RAF1* fusions are identified in approximately 20–30% of tumors, with *SND1*–*BRAF* reported as the most prevalent fusion event [13,35,43]. These rearrangements result in constitutive MAPK activation, analogous to that driven by *BRAF* V600E mutations [13].

Preclinical and clinical observations suggest that *BRAF* fusion-positive tumors may be sensitive to MEK inhibition, supporting these rearrangements as potentially actionable therapeutic targets [13,35]. Less frequent but clinically relevant fusions involving *RET*, *ALK*, NTRK, *ROS1*, and *PRKACA* have also been described [35,43,54]. Individual cases of durable disease control with matched targeted therapies have been reported. For example, one patient with *RET* fusion-positive PACC achieved disease control exceeding 42 months with selpercatinib [54]. However, these observations are based on single case reports and should not be interpreted as established evidence of efficacy; prospective validation is required.

### 4.6. Microsatellite Instability

Mismatch repair deficiency and microsatellite instability-high (MSI-H) are uncommon but reproducible findings in PACC, reported in approximately 5–10% of cases, including tumors arising in the context of Lynch syndrome [16,46]. A small subset of tumors exhibits high tumor mutational burden and may be candidates for immune checkpoint blockade [44,45]. A reported case of MSI-H PACC achieved a complete pathological response following pembrolizumab therapy after progression on prior systemic treatment, ultimately permitting curative-intent resection. This observation provides evidence that immune checkpoint inhibition may induce profound and durable responses in molecularly selected patients [45].

In contrast to PDAC, global hypermethylation is not a defining feature of PACC. Promoter hypermethylation is largely restricted to a limited number of genes, most notably *APC* and *RASSF1* [17].

**Figure 1:**
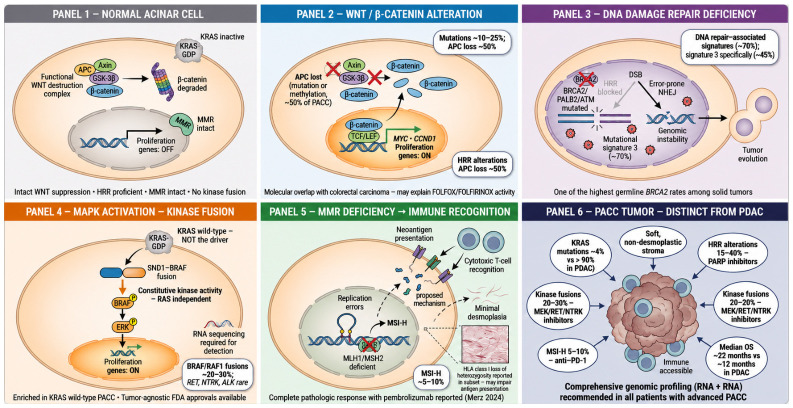
Key biological mechanisms underlying pancreatic acinar cell carcinoma (PACC) and their therapeutic implications. **Panel 1**: Normal pancreatic acinar cell with intact WNT suppression through the *APC* destruction complex, functional homologous recombination repair (HRR), no activating kinase fusion, and intact mismatch repair (MMR). **Panels 2–5** illustrate the four principal oncogenic mechanisms identified in PACC, each mechanistically distinct from the canonical *KRAS*-driven pathway of pancreatic ductal adenocarcinoma (PDAC). **Panel 2**: Loss of *APC* through chromosomal deletion (~48%) or promoter hypermethylation (~56%), with point mutations in only 7% of cases, inactivates the β-catenin destruction complex, allowing β-catenin to translocate to the nucleus and activate TCF/LEF-driven transcription of proliferation genes [15,17,18]. **Panel 3**: Germline or somatic mutations in HRR genes including *BRCA2*, *BRCA1*, *PALB2*, and *ATM*, present in approximately 15–40% of PACC cases, impair faithful double-strand break repair, forcing reliance on error-prone non-homologous end joining (NHEJ). Approximately 70% of PACCs harbor mutational signatures associated with DNA repair defects broadly, with mutational signature 3 (attributable to *BRCA1/2* loss) present in approximately 45% [35,38,40,42,44,49,50]. **Panel 4**: Chromosomal rearrangements creating oncogenic kinase fusions, most commonly *SND1*–*BRAF* and *CCDC6*–*RET*, but also involving NTRK and *ALK*, result in constitutive, RAS-independent MAPK/ERK pathway activation. *BRAF* and *RAF1* fusions account for approximately 20–30% of PACC tumors and are enriched in the *KRAS* wild-type subset. RNA-based sequencing is required for detection [13,35,43,52,53,54]. **Panel 5:** MMR deficiency leads to MSI-H status and neoantigen accumulation, enabling potential cytotoxic T-cell recognition (proposed mechanism). PACC’s minimal desmoplasia may facilitate immune access compared with PDAC. HLA class I loss of heterozygosity reported in a subset may impair antigen presentation [16,44,46]. Complete pathologic response with pembrolizumab reported in MSI-H PACC (Merz 2024) [45]. **Panel 6**: *KRAS* mutations are present in approximately 4% of PACC cases (vs. >90% in PDAC). Comprehensive genomic profiling incorporating both DNA and RNA sequencing is recommended in all patients with advanced PACC [12,13,14,35,41,44]. *APC*, adenomatous polyposis coli; β-catenin (β-cat); TCF/LEF, T-cell factor/lymphoid enhancer-binding factor; HRR, homologous recombination repair; NHEJ, non-homologous end joining; DSB, DNA double-strand break; MMR, mismatch repair; MSI-H, microsatellite instability-high; TMB, tumor mutational burden; MAPK, mitogen-activated protein kinase; ERK, extracellular signal-regulated kinase; MHC, major histocompatibility complex; pCR, pathologic complete response; PDAC, pancreatic ductal adenocarcinoma; *KRAS*, Kirsten rat sarcoma viral proto-oncogene. Created with BioRender.com.

The four principal oncogenic mechanisms described in Section 4.3, Section 4.4, Section 4.5 and Section 4.6 are illustrated in Figure 1.

## 5. Treatment and Prognosis

### 5.1. Localized Disease

A critical distinction must be made between treatment approaches that constitute standard of care for pancreatic cancer broadly and those supported by PACC-specific retrospective evidence. For localized disease, surgical resection with curative intent is established practice. Patients who undergo curative-intent resection demonstrate improved long-term survival compared with non-operative management in retrospective series, with reported median overall survival ranging from approximately 40 to 78 months in resected patients, exceeding outcomes typically observed in PDAC [1,7,55]. These comparisons are derived from non-randomized data and should be interpreted with appropriate caution. Despite resection, recurrence remains common, indicating presence of occult micrometastatic disease in nonmetastatic patients, supporting consideration of adjuvant therapy [10,11]. Recurrence most often involves the liver and regional lymph nodes. Nodal involvement at the time of resection has been consistently associated with worse disease-free and overall survival [7,11,55,56,57].

The role of adjuvant therapy in PACC remains undefined. Evidence is mixed. Some studies report overall improved outcomes, while others demonstrate a survival benefit limited to node-positive patients [56,57,58]. All available data on adjuvant therapy in PACC derive from retrospective series with significant heterogeneity in patient selection, regimens, and follow-up duration. No randomized trial data exist, and current practice is guided by extrapolation from PDAC adjuvant trials. Selected studies evaluating adjuvant and systemic therapy in PACC are summarized in Table 2.

### 5.2. Metastatic Disease

For advanced disease, there is no PACC-specific standard of care. Fluoropyrimidine- and platinum-containing regimens (e.g., fluorouracil, leucovorin, oxaliplatin, and irinotecan [FOLFIRINOX] are often favored, although not all studies have demonstrated a clear advantage over gemcitabine-based therapy, likely reflecting small cohort sizes and treatment heterogeneity [23,26,60,61,63,64]. In select patients with oligometastatic disease, metastasectomy or combined modality treatment may be associated with favorable outcomes based on small case series [19,64].

### 5.3. Targeted and Immunotherapy Approaches

Note: Therapeutic strategies in this section are based on varying levels of evidence. PARP inhibitor use is supported by a Phase 3 trial in BRCA-mutated pancreatic cancer (POLO trial [50]), though not specifically in PACC. All other approaches are extrapolated from other tumor types or derived from isolated PACC case reports.

- Homologous Recombination Deficiency (HRD): Tumors with HRR gene alterations including *BRCA1/2* may respond favorably to PARP inhibitors by extrapolation from BRCA-mutated metastatic pancreatic cancer data (POLO trial), with very limited PACC-specific evidence [50].
- MAPK Pathway–Driven Tumors: Recurrent *BRAF* and *RAF1* fusions define a MAPK-driven subset, which has shown sensitivity to MEK inhibition [13,35].
- Other Targetable Kinase Fusions: Rare rearrangements involving NTRK, *RET*, *ALK*, and *ROS1* have been described. Because several of these alterations have tumor-agnostic therapy approvals, comprehensive genomic profiling is critical in advanced disease [35,43,54].
- Immunotherapy: Mismatch repair deficiency and MSI-H are identified in approximately 5–10% of cases, and a small subset of tumors demonstrates high tumor mutational burden [16,46]. These features may predict responsiveness to immune checkpoint blockade, although evidence specific to PACC remains limited [62]. Additional support for immunotherapy comes from a recent case of high TMB PACC treated with toripalimab and bevacizumab following chemotherapy failure. Significant tumor regression enabled subsequent R0 resection, with durable recurrence-free survival reported after surgery. These findings suggest that TMB may serve as a clinically relevant biomarker for immunotherapy selection in PACC [65].

### 5.4. Immune Crosstalk in PACC

Dedicated characterization of the immune microenvironment in PACC remains limited, representing a significant gap in the literature. Mandelker et al. identified HLA class I loss of heterozygosity in a substantial proportion of PACC tumors, suggesting that antigen presentation may be impaired in a subset of cases regardless of neoantigen burden [44]. PD-L1 expression has been reported in individual cases [62], and complete pathologic response to pembrolizumab has been documented in MSI-H PACC [45], providing proof-of-concept for immune engagement in molecularly selected patients. Systematic studies evaluating PD-L1 expression, tumor-infiltrating lymphocyte density, and immune cell composition across PACC tumors are needed to define the immune landscape of this disease and identify predictive biomarkers beyond MSI and TMB.

### 5.5. Practical Approach to Treatment Selection

Given the absence of prospective trial data, treatment selection in PACC requires integration of clinical stage, performance status, and molecular profiling results. For localized resectable disease, surgical resection with curative intent remains the primary treatment. Adjuvant chemotherapy may be considered particularly in node-positive patients, with fluoropyrimidine- or platinum-containing regimens preferred based on available retrospective data [55,56,57,58]. For advanced or metastatic disease, comprehensive genomic profiling should be obtained prior to initiating systemic therapy. In the absence of an actionable alteration, fluoropyrimidine- and platinum-containing regimens (FOLFIRINOX) are preferred based on retrospective evidence suggesting superior outcomes compared with gemcitabine monotherapy [60,61,63,64,65,66]. When actionable alterations are identified, matched targeted therapy should be considered: PARP inhibitors for HRR-deficient tumors, MEK inhibitors for *BRAF*/*RAF1* fusion-positive tumors, and approved kinase inhibitors for *RET*, NTRK, or *ALK* fusion-positive tumors per tumor-agnostic approvals. Given the available evidence, routine testing for MSI status and tumor mutational burden should be considered in all patients with advanced PACC where tissue and resources permit, as these biomarkers may identify candidates for immune checkpoint blockade and inform clinical trial eligibility. Clinical trial enrollment should be actively pursued in all settings.

### 5.6. Prognosis Summary

PACC exhibits a clinical course distinct from PDAC and is generally associated with better overall survival [2,20,55,66,67]. Outcomes are substantially worse in metastatic disease than in localized disease [63,67]. However, long-term cure is uncommon, and high recurrence rates following resection underscore the likelihood of occult micrometastatic disease and the need for improved systemic therapy strategies [10,11].

## 6. Conclusions

PACC represents a distinct pancreatic malignancy with unique clinical and molecular features that differentiate it from PDAC. Although surgical resection remains the cornerstone of treatment for localized disease, recurrence rates remain high, particularly among patients with node-positive or advanced-stage tumors. Outcomes in metastatic disease are variable, and standardized systemic treatment recommendations remain limited.

Advances in molecular profiling have substantially improved our understanding of PACC biology. The relative absence of *KRAS* mutations and the enrichment of alterations in DNA repair pathways, WNT signaling, and gene fusions underscore its distinct pathogenesis and create actionable therapeutic opportunities. Nevertheless, robust prospective data remain limited.

### Limitations

Several limitations of the available evidence merit acknowledgment. All existing studies of PACC are retrospective; no prospective randomized controlled trials have been conducted for any aspect of management. Published series are typically small, limiting statistical power and generalizability, and selection bias is inherent given the concentration of cases at academic centers. Comparison of outcomes across studies is further confounded by heterogeneous regimen definitions, variable inclusion of mixed acinar histology, and differing staging criteria. The absence of a consensus molecular profiling protocol means that reported genomic frequencies vary depending on whether DNA-only or combined DNA and RNA sequencing was performed, with the latter essential for fusion detection.

Several critical questions remain unresolved. Whether PACC management should continue to be extrapolated from PDAC or warrants a fully independent evidence base can only be answered through disease-specific prospective trials. The clinical relevance of specific alterations such as chromosomal instability and *APC* methylation remains uncertain, and the role of neoadjuvant therapy in locally advanced PACC is entirely unstudied. Addressing these gaps will require international collaboration, centralized biobanking, and inclusion of PACC patients in basket and biomarker-driven trials. Comprehensive genomic profiling to identify DDR/HRR alterations and kinase fusions should be considered standard practice in all patients with advanced PACC, as actionable alterations are identified in a substantial proportion of cases and directly inform treatment selection and trial eligibility.

## Figures and Tables

**Table 1:** Potentially Actionable Molecular Alterations in Pancreatic Acinar Cell Carcinoma. This table summarizes the principal actionable alterations in PACC and their potential therapeutic strategies, ranging from platinum agents and PARP inhibitors for HRR-deficient tumors to kinase inhibitors for fusion-positive tumors and immune checkpoint blockade for MSI-H or high TMB tumors. Evidence is largely extrapolated from other tumor types or derived from case reports and small series.

Pathway/Alteration	Approx. Frequency in PACC	Mechanistic Rationale	Targeted Strategy	Evidence Base	Key Citations
*APC*–β-catenin pathway alterations (*APC* mutation/loss, *CTNNB1* mutation, *APC* hypermethylation)	10–25% (*APC*/*CTNNB1* mutations); up to ~50% *APC* loss/methylation	Constitutive Wnt/β-catenin activation; molecular overlap with colorectal carcinoma type signaling	Consideration of colorectal-type regimens (5-FU, irinotecan, oxaliplatin); investigational Wnt pathway inhibitors	Retrospective series; biological rationale by analogy with colorectal cancer	[15,16,17,18,19]
*BRCA1/2*, *PALB2* (HRR genes)	15–40%	Homologous recombination deficiency; defective double-strand DNA repair	Platinum-based chemotherapy; PARP inhibitors	Extrapolated from BRCA-mutated PDAC (POLO trial); limited PACC-specific data	[35,38,40,41,42]
*BRAF* fusion–positive tumors (e.g., *SND1*–*BRAF*)	20–30%	Constitutive MAPK pathway activation independent of *KRAS*	MEK inhibitors ± *BRAF*-directed therapy	Preclinical data; mixed clinical responses in isolated PACC case reports; pan-cancer basket trial rationale	[13,35,43]
High Tumor Mutational Burden (high TMB)	Rare subset	Increased neoantigen burden and immunogenicity	Immune checkpoint inhibitors (PD-1 blockade)	FDA tumor-agnostic approval for MSI-H (pembrolizumab); one complete response in MSI-H PACC; one case report in high TMB PACC	[44,45]

**Table 2:** Selected studies evaluating adjuvant and systemic therapy in pancreatic acinar cell carcinoma (PACC). All studies are non-randomized retrospective analyses. Sample sizes (*n*) and country of origin are provided for all studies to contextualize the strength of evidence. Findings should be interpreted with caution given the small cohort sizes, treatment heterogeneity, and absence of prospective data.

Study (Year)	Study Design/Cohort	Setting	Treatment Evaluated	Key Findings	Conclusions
Patel et al., 2020 [59]	National Cancer Database (NCDB) retrospective analysis (*n* = 298, USA)	Clinically resectable PACC	Surgical resection versus surgical resection with adjuvant chemotherapy	Adjuvant chemotherapy was associated with improved overall survival compared with surgery	Supports consideration of adjuvant chemotherapy after resection
Petrova et al., 2021 [55]	German Cancer Registry Group retrospective analysis (*n* = 233 full cohort, 127 matched, Germany)	Mixed stages PACC and PDAC	Surgery ± adjuvant therapy	No clear survival benefit of adjuvant therapy in PACC; benefit observed in matched PDAC cohort	Surgical resection is the primary independent positive prognostic factor and should be advocated even in advanced tumor stages. No survival benefit demonstrated for adjuvant therapy in PACC
Seo et al., 2017 [7]	Single-institution retrospective (*n* = 20, South Korea)	Clinically resectable PACC	Surgery ± adjuvant therapy	Resectable PACC has better OS than PDAC after surgery	Favorable prognosis versus PDAC after surgery; role of adjuvant chemotherapy remains undefined
Schmidt et al., 2008 [11]	NCDB retrospective analysis (*n* = 865, USA)	Mixed stages PACC and PDAC	Surgery ± adjuvant therapy	PACC carries a better prognosis than PDAC; Surgical resection associated with improved survival	Surgery is cornerstone, especially in negative margins
Sridharan et al., 2021 [60]	Single-institution retrospective (*n* = 66, USA)	Mixed stages PACC	Surgery, chemotherapy	Systemic therapy (particularly platinum- and fluoropyrimidine-based regimens) showed activity in advanced disease	Chemotherapy beneficial in metastatic/unresectable PACC
Takahashi et al., 2021 [61]	Multicenter retrospective (*n* = 58, Japan)	Advanced/metastatic PACC	Various systemic regimens	Platinum-and irinotecan containing regimens demonstrated higher response rates and longer survival than those who did not receive either	Platinum-and irinotecan-containing regimens may be preferred in advanced PACC
Woo et al., 2024 [56]	NCDB retrospective analysis (*n* = 1553, USA)	Mixed stages PACC	Surgical resection versus surgical resection with adjuvant chemotherapy	Surgical resection remains primary effective treatment; adjuvant chemotherapy benefit more pronounced in node-positive patients	Adjuvant chemotherapy is associated with improved OS only in node-positive cases
Xu et al., 2021 [62]	Single-institution retrospective (*n* = 22, China)	Advanced/metastatic	Fluoropyrimidine vs. gemcitabine-based regimens	Fluoropyrimidine-based therapy associated with improved PFS and OS compared with gemcitabine	Supports preference for fluoropyrimidine-based regimens
Yoo et al., 2017 [63]	Single-institution retrospective (*n* = 15, South Korea)	Advanced/metastatic	Various systemic regimens	Higher response rates observed with oxaliplatin-based combinations; gemcitabine monotherapy showed limited efficacy	Oxaliplatin-based chemotherapy appears more effective

## Data Availability

No new data were created or analyzed in this study. Data sharing is not applicable to this article.
